# Preoperative predictors of early death risk in bladder cancer patients treated with robot‐assisted radical cystectomy

**DOI:** 10.1002/cam4.2237

**Published:** 2019-05-15

**Authors:** Zhaowei Zhu, Xiaojing Wang, Jiange Wang, Shengzheng Wang, Yafeng Fan, Tianlong Fu, Songqiang Cao, Xuepei Zhang

**Affiliations:** ^1^ Department of Urology The First Affiliated Hospital of Zhengzhou University Zhengzhou PR China; ^2^ Department of Urology Ruijin Hospital, Shanghai Jiao Tong University School of Medicine Shanghai China; ^3^ Department of Urology Zhengzhou Central Hospital Zhengzhou PR China; ^4^ Department of Urology Huaihe Hospital of Henan University Kaifeng PR China

**Keywords:** risk assessment, surgical oncology, survival, urological oncology

## Abstract

**Background:**

Early identification of early death for bladder cancer patients undergoing radical cystectomy based on the laboratory findings at the time of diagnosis could improve the overall survival. The study aimed to explore preoperative factors associated with higher risk of early death (within 1 year after surgery) for bladder cancer patients.

**Methods:**

A total of 186 bladder cancer patients who underwent robot‐assisted radical cystectomy (RARC) were identified between October 2014 and May 2017. The probability of dying within 1 year after RARC was defined as the end point “early death.” Predictive factors including clinical features and laboratory findings at diagnosis were retrospectively collected.

**Results:**

Median follow‐up time after RARC was 20.6 months (1.2‐43.7 months). Fifty‐one patients (27.4%) died during follow‐up and 31 within 1 year from surgery (1‐year mortality rate: 16.7%). All potentially prognostic factors were assessed on univariate analyses, which revealed the following factors as being associated with higher risk of early death within 1 year after RARC: older age (*P* = 0.004), advanced clinical stage (*P* = 0.005), presence of hydronephrosis (*P* = 0.021), higher fibrinogen (*P* = 0.007), higher PLR (*P* = 0.031), and lower PNI (*P* = 0.016). In a multivariate Cox proportional hazard regression model analysis, age >60 years (HR = 7.303, 95% CI 1.734‐30.764; *P* = 0.007) and fibrinogen ≥3.295 g/L (HR = 2.396, 95% CI 1.138‐5.045; *P* = 0.007) at diagnosis were independent prognostic factors of early death after RARC.

**Conclusion:**

Age and preoperative elevated plasma fibrinogen level were independent predictors for 1‐year mortality after RARC. We believe that plasma fibrinogen levels may become a useful biomarker, which may help guide the treatment decision‐making process for patients with bladder cancer.

## 1 INTRODUCTION

Bladder carcinoma represents the first and second most common urological malignancy in China and USA, respectively.^1^, ^2^ Radical cystectomy is the main treatment modality for muscle‐invasive and high‐risk nonmuscle‐invasive urothelial carcinoma of the bladder.^3^ Although open surgery remains the most commonly adopted surgical approach, robot‐assisted radical cystectomy (RARC) has evolved over the last few years to become an acceptable option for patients with bladder cancer.

With advances in da Vinci robot systems and surgical skills, three‐year overall survival (OS) rates after RARC have reached 61%‐80%.^4^ Noteworthy, some bladder cancer patients still die within the first year from RARC. The preoperative identification of these patients has a strong clinical value, since these patients can be selected for neoadjuvant chemotherapy rather than upfront surgery. However, it is difficult to identify patients who are at high risk of early mortality due to the poor prognostic value of the traditional TNM staging system.

Previous researches have identified various predictors of early mortality among patients receiving radical cystectomy, but few of them included only preoperative factors.^5^, ^6^, ^7^ The objective of this study was to explore preoperative predictors associated with higher risk of early death (within 1 year after RARC) for bladder cancer patients.

## 2 METHODS

We retrospectively reviewed 186 patients with bladder cancer who underwent RARC between October 2014 and May 2017 in our institution. No bladder cancer patients received neoadjuvant chemotherapy before RARC. The primary objective was the identification of preoperative predictors of early death, which were defined as death within 1 year of RARC.^8^ The following variables were studied: patient age, gender, American Societyof Anesthesiologists (ASA) score, body mass index (BMI), no. of tumors, preoperative clinical stage, and hydronephrosis. Preoperative laboratory evaluation should include complete blood count, fibrinogen, and liver function testing.

The endpoint of the study was OS, which was calculated from the day of surgery to the time of any‐cause mortality, or was censored at the date of recent follow‐up.^9^, ^10^ PNI was calculated as albumin level (g/L) + 5 × lymphocyte count (10^9^/L),^10^ NLR as neutrophil/lymphocyte count, PLR as platelet/lymphocyte ratio, LMR as lymphocyte/monocyte count, and AGR as albumin/(total protein‐albumin).^10^ Receiver operating characteristic (ROC) curve analysis was used to compare the prognostic ability of each indicator for each OS event according to the area under the ROC curve (AUC) and to determine the best cutoff points.^10^ Patients were classified into two categories according to the cutoff values.

Cox univariate analysis was used to identify prognostic factors for predicting 1‐year OS, estimating hazard ratios (HRs), and 95% confidence intervals (CIs). Factors significant on univariate analysis were included in Cox proportional‐hazards multivariate models.^10^ Analyses were performed using SPSS version 20.0 (SPSS, Chicago, IL). All tests were two‐sided and a *P* < 0.05 was considered statistically significant.

## 3 RESULTS

A total of 186 patients were included in this study. The clinicopathological characteristics of all patients are shown in Table 1. Median patient age was 65 years (IQR 32 to 86), 66.1% of the patients were older than 60 years of age, 84.4% of the patients were men, 14.0% had ASA score ≥3, 47.3% had multiple tumors and 26.3% had hydronephrosis. The tumor stage of all patients was ≤T1 for 39 (21.0%), T2 for 125 (67.2%), T3 or higher for 22 (11.8%). Median fibrinogen was 3.13 (IQR 1.22‐6.26), NLR: 2.62 (IQR 0.63‐34.5), PLR: 129.13 (IQR 34.55‐830.00), LMR: 3.33 (IQR 0.34‐8.28), AGR: 1.58 (IQR 0.73‐2.97), PNI: 47.0 (IQR 27.2‐63.1).

**Table 1:** Patient demographics and clinical characteristics of bladder cancer patients who received RARC

Variable	All patients, N = 186
Age, years, median (IQR)	65 (32‐86)
Gender, n (%)	
Male	157 (84.4)
Female	29 (15.6)
BMI, kg/m^2^, median (IQR)	24.7 (16‐45)
ASA score ≥3, n (%)	26 (14.0)
No. of tumors, n (%)	
Solitary	98 (52.7)
Multiple	88 (47.3)
Hydronephrosis, n (%)	
Absent	137 (73.7)
Unilateral	34 (18.3)
Bilateral	15 (8.1)
Clinical stage, n (%)	
≤T1	39 (21.0)
T2	125 (67.2)
≥T3	22 (11.8)
Pathological type, n (%)	
UC	176 (94.6)
NUC	10 (5.4)
Pathological grade, n (%)	
PUNLMP	8 (4.3)
LGPUC	62 (33.3)
HGPUC	90 (48.4)
Fibrinogen, median (IQR)	3.13 (1.22‐6.26)
NLR, median (IQR)	2.62 (0.63‐34.5)
PLR, median (IQR)	129.13 (34.55‐830.00)
LMR, median (IQR)	3.33 (0.34‐8.28)
AGR, median (IQR)	1.58 (0.73‐2.97)
PNI, median (IQR)	47.0 (27.2‐63.1)

Abbreviations: AGR, albumin‐globulin ratio; ASA, American Society of Anesthesiologists; BMI, body mass index; HGPUC, high‐grade papillary urothelial carcinoma; IQR, interquartile range; LGPUC, low‐grade papillary urothelial carcinoma; LMR, lymphocyte‐monocyte ratio; NLR, neutrophil‐lymphocyte ratio; NUC, non‐urothelial carcinoma; PLR, platelet‐lymphocyte ratio; PNI, prognostic nutritional index; PUNLMP, papillary urothelial neoplasm of low malignant potential; UC, urothelial carcinoma.

**Figure 1:**
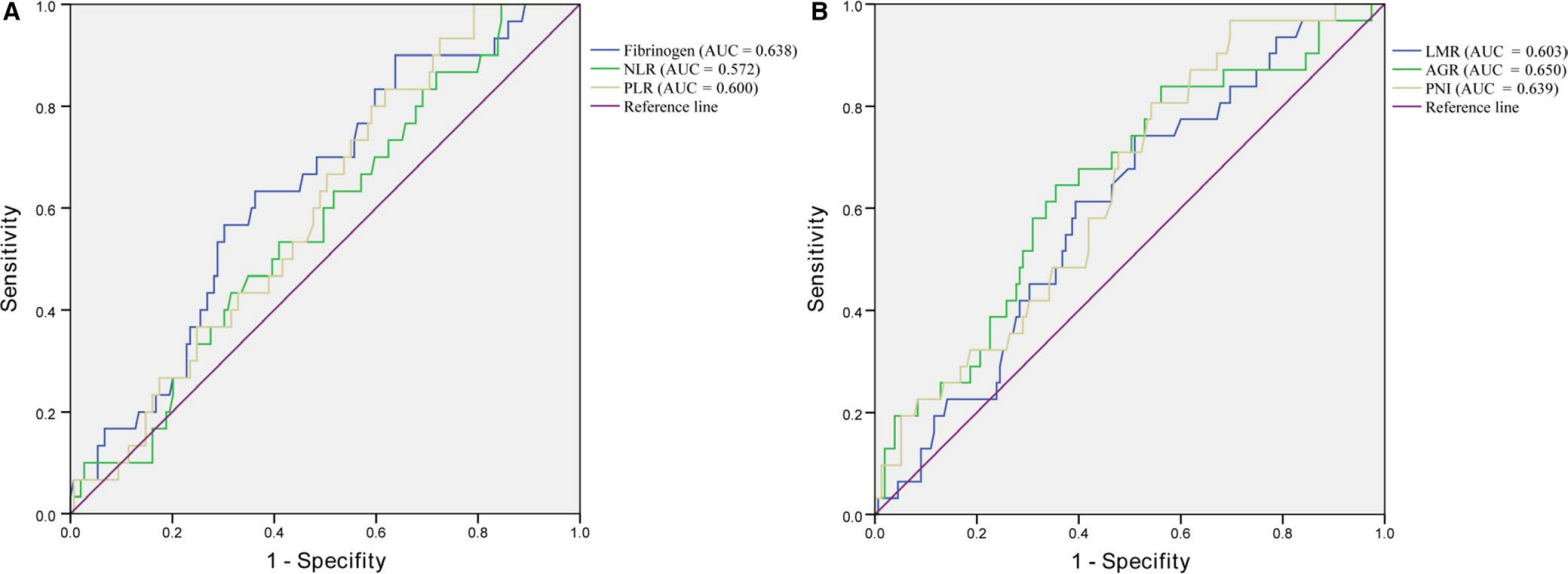
Receiver operating characteristic (ROC) curves for overall survival

We determined the cutoff values for the six factors for OS by calculating the maximum Youden index (fibrinogen‐3.295, NLR‐1.4388, PLR‐111, LMR‐3.4974, AGR‐1.5262, PNI‐50.95). Then patients were divided into low‐ and high‐risk groups according to the cutoff points. The AUC value was greater for fibrinogen than NLR and PLR for estimating OS (Figure 1).

Median follow‐up time after RARC was 20.6 months (1.2‐43.7 months). Fifty‐one patients (27.4%) died during follow‐up and 31 (16.7%) within 1 year from surgery. Table 2 compares the preoperative risk factors for postoperative death within 1 year after RARC. One‐year mortality was significantly associated with age, clinical stage, fibrinogen, NLR, PLR, and PNI.

**Table 2:** Comparison of preoperative risk factors for postoperative death within 1 year after RARC

Variables	Death within 1 year (31)	Death after 1 year (155)	*P* value
Age, years, median (IQR)	69 (51‐86)	63 (32‐83)	<0.001
Gender, n (%)			
Male	26 (83.9)	131 (84.5)	1.000
Female	5 (16.1)	24 (15.5)	
BMI, kg/m^2^	23.31 (18‐32)	25.10 (16‐45)	0.070
ASA score ≥3, n (%)	6 (19.4)	20 (12.9)	0.499
No. of tumors, n (%)			
Solitary	20 (64.5)	78 (50.3)	0.171
Multiple	11 (35.5)	77 (49.7)	
Clinical stage, n (%)			
≤T1	2 (6.5)	37 (23.9)	0.001
T2	20 (64.5)	105 (67.7)	
≥T3	9 (29.0)	13(8.4)	
Pathological type, n (%)			
UC	30 (96.8)	146 (94.2)	1.000
NUC	1 (3.2)	9 (5.8)	
Pathological grade, n (%)			
PUNLMP	0 (0.0)	8 (5.9)	0.338
LGPUC	8 (33.3)	54 (39.7)	
HGPUC	16 (66.7)	74 (54.4)	
Hydronephrosis, n (%)			
Absent	18 (58.1)	119 (76.8)	0.068
Unilateral	8 (25.8)	26 (16.8)	
Bilateral	5 (16.1)	10 (6.5)	
Fibrinogen, n (%)			
<3.295	11 (36.7)	95 (63.8)	0.008
≥3.295	19 (63.3)	54 (36.2)	
NLR, n (%)			
<1.4388	0 (0.0)	24 (15.5)	0.016
≥1.4388	31 (100.0)	131 (84.5)	
PLR, n (%)			
<111	5 (16.1)	58 (37.4)	0.023
≥111	26 (83.9)	97 (62.6)	
LMR, n (%)			
<3.4974	2 (6.5)	14 (9.0)	1.000
≥3.4974	29 (93.5)	141 (91.0)	
AGR, n (%)			
<1.5262	28 (90.3)	134 (86.5)	0.771
≥1.5262	3 (9.7)	21 (13.5)	
PNI, n (%)			
<50.95	25 (80.6)	87 (56.1)	0.015
≥50.95	6 (19.4)	68 (43.9)	

Abbreviations: AGR, albumin‐globulin ratio; ASA, American Society of Anesthesiologists; BMI, body mass index; HGPUC, high‐grade papillary urothelial carcinoma; IQR, interquartile range; LGPUC, low‐grade papillary urothelial carcinoma; LMR, lymphocyte‐monocyte ratio; NLR, neutrophil‐lymphocyte ratio; NUC, non‐urothelial carcinoma; PLR, platelet‐lymphocyte ratio; PNI, prognostic nutritional index; PUNLMP, papillary urothelial neoplasm of low malignant potential; UC, urothelial carcinoma.

**Figure 2:**
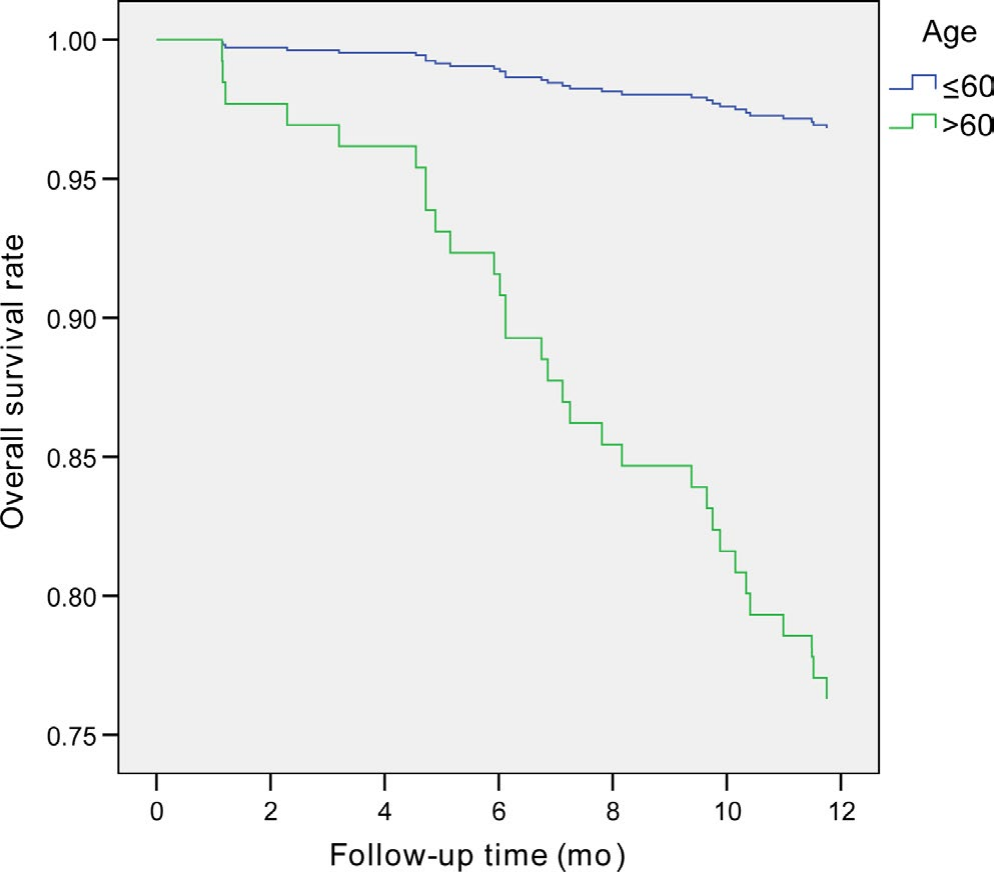
Comparison of survival curves of bladder cancer patients at different ages

**Figure 3:**
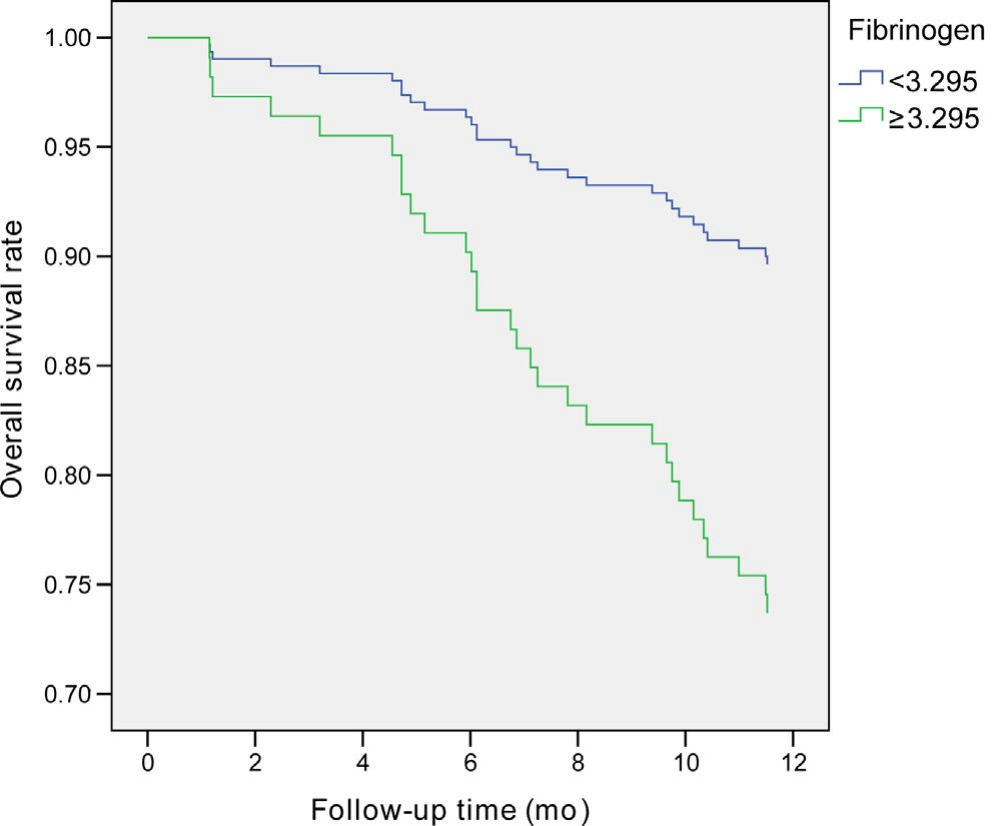
Comparison of survival curves of bladder cancer patients with different preoperative plasma fibrinogen levels

In Cox univariate analysis, six prognostic variables were associated with higher risk of early death within 1 year after RARC: older age (*P* = 0.004), advanced clinical stage (*P* = 0.005), presence of hydronephrosis (*P* = 0.021), higher fibrinogen (*P* = 0.007), higher PLR (*P* = 0.031) and lower PNI (*P* = 0.016) (Table 3). On multivariate Cox stepwise analysis, older age (>60 years: HR = 7.303, 95% CI 1.734‐30.764; *P* = 0.007) (Figure 2) and higher fibrinogen (≥3.295 g/L: HR = 2.396, 95% CI 1.138‐5.045; *P* = 0.007) (Figure 3) remained independently associated with 1‐year mortality after RARC (Table 4).

**Table 3:** Preoperative prognostic factors for 1‐year mortality after RARC according to univariate COX regression

Variables	HR	95%CI	*P* value
Age (>60 vs ≤60)	8.37	2.00‐35.08	0.004
Gender (male vs female)	0.959	0.368‐2.496	0.931
BMI (≥22.6 vs <22.6)	0.542	0.267‐1.099	0.089
ASA score (≥3 vs <3)	1.526	0.626‐3.720	0.353
No. of tumors (multiple vs solitary)	0.599	0.287‐1.251	0.173
Clinical stage (vs. ≤T1)			
T2	3.264	0.763‐13.966	0.111
≥T3	9.217	1.990‐42.690	0.005
Pathological type (NUC vs UC)	0.568	0.077‐4.163	0.578
Pathological grade (HGPUC vs LGPUC)	1.372	0.587‐3.207	0.465
Hydronephrosis (present vs absent)	1.714	1.086‐2.706	0.021
Fibrinogen (high vs low)	2.787	1.326‐5.858	0.007
NLR (high vs low)	25.360	0.308‐2088.523	0.151
PLR (high vs low)	2.876	1.104‐7.490	0.031
LMR (high vs low)	1.363	0.325‐5.711	0.672
AGR (high vs low)	0.674	0.205‐2.218	0.517
PNI (low vs high)	3.002	1.231‐7.318	0.016

Abbreviations: AGR, albumin‐globulin ratio; ASA, American Society of Anesthesiologists; BMI, body mass index; HGPUC, high‐grade papillary urothelial carcinoma; IQR, interquartile range; LGPUC, low‐grade papillary urothelial carcinoma; LMR, lymphocyte‐monocyte ratio; NLR, neutrophil‐lymphocyte ratio; NUC, non‐urothelial carcinoma; PLR, platelet‐lymphocyte ratio; PNI, prognostic nutritional index; PUNLMP, papillary urothelial neoplasm of low malignant potential; UC, urothelial carcinoma.

**Table 4:** Preoperative prognostic factors for 1‐year mortality after RARC by multivariate cox regression analysis

Variables	Adverse factor	HR	95% CI	*P* value
Age (years)	>60	7.303	1.734‐30.764	0.007
Clinical stage (vs ≤T1)	T2			0.669
	≥T3			0.077
Hydronephrosis (vs absent)	present			0.217
Fibrinogen (g/L)	≥3.295	2.396	1.138‐5.045	0.021
PLR (vs low)	High			0.196
PNI (vs high)	Low			0.150

Abbreviations: CI, confidence interval; HR, hazard ratio; PLR, platelet‐lymphocyte ratio; PNI, prognostic nutritional index.

## 4 DISCUSSION

Our goal was to identify preoperative risk factors predicting 1‐year mortality after RARC. Such early death might be caused by underestimation of tumor stage, aggressive characteristic of bladder cancer, or perioperative complications.^11^ We found that older age and higher plasma fibrinogen levels were individual predictors in the multivariate models. This may guide the urologist in oncologic outcome prediction and in choosing eligible patients who might benefit from adjuvant systemic therapy.

Traditionally, prognostic factors for bladder cancer have been focusing on tumor stage and histological grade. Noteworthy, older patients have more years to compound comorbidities which may influence survival of bladder cancer patients. It is unclear whether patient age is an independent prognostic factor for patients undergoing RARC and there is still much discrepancy in previous studies. Some studies have shown that age is associated with poor survival^12^, ^13^, ^14^ while others have suggested that it is not a prognostic factor in multivariate analysis.^15^, ^16^, ^17^ Although experienced surgeons could safely carry out RARC in elderly patients,^17^, ^18^ the present findings suggested that elderly patients (＞60 years) have worse OS compared with younger patients (≤60 years) undergoing RARC.

Many patients with bladder cancer were found to have hydronephrosis due to ureteral orifice obstruction by tumor. Although pathologic stage is regarded as crucial prognostic characteristic after radical cystectomy, the prognostic role of hydronephrosis has been investigated with conflicting results.^19^, ^20^, ^21^, ^22^ To the best our knowledge, the relationship between the presence of preoperative hydronephrosis and prognosis after RARC has not yet been investigated. In our study, we found that preoperative hydronephrosis was significantly associated with 1‐year mortality after RARC according to univariate cox regression. This could assist in assessing the prognosis of patients and deciding therapeutic plans preoperatively.

Increasing studies shows that an interactive relationship exists between haemostatic factors and tumor biology.^23^, ^24^ Fibrinogen, one of the haemostatic factors, is a plasmaglyco protein whick plays a key role in clot formation and wound healing.^24^ Previous studies have demonstrated that elevated pre‐therapeutic plasma fibrinogen levels are associated with worse outcome in patients with ovarian or endometrial cancer.^25^, ^26^

Tanaka et al demonstrated that preoperative plasma fibrinogen level was an independent prognostic variable for poor survival after radical nephroureterectomy in patients with upper tract urothelial carcinoma.^24^ Liu and colleagues observed that elevated preoperative plasma fibrinogen level was an independent predictor of advanced clinical stage in patients with bladder cancer.^27^ However, the prognostic value of plasma fibrinogen levels for bladder cancer patients receiving radical cystectomy has not yet been reported. We believe that preoperative plasma fibrinogen levels might become a useful biomarker in clinical practice.

However, some limitations of the present study warrant mention. First, the study was limited by the inherent drawbacks of its retrospective design. Second, this study was a single, tertiary‐care institution study. Finally, the number of patients is somewhat small to make a final conclusion on this matter. Thus, we are conducting a prospective randomized study to confirm the results in real clinical practice.

## 5 CONCLUSIONS

In summary, age and preoperative plasma fibrinogen level are independent predictors for 1‐year mortality after RARC. To the best of our knowledge, this is the first study to investigate the prognostic value of preoperative plasma fibrinogen levels in bladder cancer patients treated with RARC. We believe that preoperative plasma fibrinogen levels might become a useful biomarker for innovative systemic therapy aiming at improving prognosis in these patients.

## CONFLICT OF INTEREST

The authors declare that they have no conflicts of interest.
